# Body Image Concerns: The Impact of Digital Technologies and Psychopathological Risks in a Normative Sample of Adolescents

**DOI:** 10.3390/bs12080255

**Published:** 2022-07-27

**Authors:** Martina Mesce, Luca Cerniglia, Silvia Cimino

**Affiliations:** 1 Department of Dynamic, Clinical and Health Psychology, University of Rome, Sapienza, 00186 Rome, Italy; mesce.1765142@studenti.uniroma1.it (M.M.); silvia.cimino@uniroma1.it (S.C.); 2 Faculty of Psychology, International Telematic University Uninettuno, 00186 Rome, Italy

**Keywords:** body image concerns, social media addiction, internet addiction, adolescence

## Abstract

Background and Objectives: Previous research on associations between Body Image Concerns (BIC) and technological addictions, such as Internet addiction (IA) and Social Media Addiction (SMA), has focused on female samples, neglecting the impact they may have on males and the risk factor associated with age. The present study analyzed the correlations between BIC, IA, and SMA and between internalizing and externalizing problems. Methods: A sample of 204 participants (118 females; mean age = 15.88 years) were divided into three age groups (early, middle, and late adolescence) and completed a battery of scales including (i) Body Image Concern Inventory, (ii) Bergen Instagram Addiction Scale, (iii) Internet Addiction Test, and (iv) Youth Self Report. Results: Significant associations between BIC and technology addictions (SMA and IA) appeared both in the total sample and in the subgroups related to gender and age; bivariate correlations between internalizing and externalizing problems and variables were significant for the total sample but only in some of the gender- and age-related subgroups. Discussion and Conclusions: This research has shown how associations between BIC and behavioral technology addictions, especially associations with internalizing and externalizing symptoms, may vary by the gender affiliation and developmental stage of the individual.

## 1. Introduction

### 1.1. Theoretical Background

One of the most striking features of this era is the rapid spread of new media, which has led to the development of entirely new means of staying in touch and engaging with others. This has caused profound changes in public and private life, bringing benefits as well as potentially dangerous conditions; the latter include reduced social interaction with friends and family, loneliness, alienation, disputes in social relationships, a decline in school performance, and even mental illness [1,2,3]. Excessive internet use can become a risk factor and cause addiction [4]. Prensky [5] coined the term “digital natives” to refer to the period from preadolescence to young adulthood, as these individuals were born into a world already accustomed to technology; when compounded by the vulnerability typical of this stage of life, this makes this age group the most affected by the technological revolution. In fact, it has been shown [6] that it is during these years that individuals undergo profound changes involving physiological, psychosocial, temporal, and cultural aspects, and furthermore, it is during this period that they are most exposed to cultural ideals of beauty. Indeed, sociocultural factors play a key role as they influence biological factors, conditioning the course of adolescence, identity formation, and the attention young people pay to their physical appearance. As a result, these factors can alter an adolescent’s self-esteem and perception of his or her body [7]; the young people thus find themselves reorganizing their representations of self and body [8].

The internet and social media provide a new space where adolescents can explore and express themselves and become familiar with new body characteristics, and where they can accept and integrate them with the support of peers [9]. Sociocultural theories of body image, such as the tripartite model [10], argue that social media can condition the perception of body image because they emphasize the importance of appearance and drive the achievement of unrealistic body ideals through mediating mechanisms such as internalization and comparison that underlie body dissatisfaction [11].

In fact, objectification theory [12,13] suggests that the mediation of body surveillance plays an important role in the relationship between media internalization and body dissatisfaction; according to this theory, those who have internalized the ideals proposed by the media tend to habitually check the appearance of their bodies to make sure they match their internalized ideal. This usually results in a perceived discrepancy between the body ideal internalized by the media and one’s actual body, leading to body dissatisfaction. In fact, it has been observed [14] that when an adolescent’s body does not coincide with his or her ideal or does not reflect what is considered culturally ideal, the adolescent experiences discomfort, disillusionment, and loss. In such cases, the internet and social media provide an opportunity to present oneself to others in the closest way to what is considered ideal or desirable through the manipulation of one’s image.

In recent years, there has been growing interest in associations between social media and body-related variables (e.g., body image, body dissatisfaction, and body esteem). In particular, the literature (e.g., [15,16]) suggests that frequent engagement with social networking sites, especially platforms focused primarily on image (including Facebook and Instagram), reinforces the idea of thinness.

It has been observed that while traditional media (e.g., television and magazines) show images of celebrities and role models, social media mainly presents content from peers [17,18], and research has suggested that peer comparison is the most influential type of social comparison. In particular, comparison with peer appearance has been observed [19] to mediate the effects of social media activities (e.g., browsing and editing photos on Instagram) on body esteem.

It is important to note that the extent of social media’s influence on body image is the same for girls and boys [20,21], and that because of the stigma attached to male body image, the impact of social media on boys may be underestimated because boys usually tend to downplay body image issues [22].

Boys and girls may face different body-related challenges and pressures on social media [23,24], and may consequently use different strategies to manage these pressures. Indeed, the concerns expressed by girls typically focus on achieving an ideal of thinness, whereas boys’ ideal of appearance tends to be masculinity [25]. Similarly, boys tend to place more value on their functional abilities (e.g., physical qualities and strength), while girls tend to invest more in the aesthetic qualities of their outward appearance [26].

### 1.2. The Present Study

In light of these premises and of the continued growth of the virtual world and the persistent interest shown by adolescents, the present research aims to shed light on the possible coexistence of misperception of one’s body and behavioral addictions at specific developmental stages. The previous literature (e.g., [21,27,28]) has pointed out that the strong presence of appearance-related content on the internet and excessive use of social networks could be a new predictor of body dissatisfaction. Without establishing directional relationships and following research methods previously used in the literature, the purpose of this study was to investigate the existence of correlations between body image concerns (BIC) and two technology-based addictions, namely, internet addiction (IA) and social media addiction (SMA).

In addition, we wanted to observe the possible presence of psychopathological risks in adolescents, distinguished into internalizing problems (i.e., mainly causing internal difficulties in the individual’s functioning, such as depression or anxiety) and externalizing problems (i.e., mainly causing external difficulties, such as conduct problems or aggression) [29,30].

Special attention was paid to gender, which is already considered a socio-anagraphic risk factor; according to previous findings, there are differences between males and females in technology addiction [31,32,33]. Although previous studies [34,35] have already considered age as a variable, to our knowledge the present study is the first to consider this variable in the context of possible correlation between body image preoccupation and technological addictions (IA and SMA). For this reason, the sample was divided into three subgroups by age: early (14–15 years), middle (16–17 years), and late adolescence (18–19 years), following Steinberg’s suggestions [36].

## 2. Materials and Methods

### 2.1. Participants and Procedure

A total of 204 adolescents from southern Italy were recruited to take part in this study. The sample consisted of *n* = 118 females (57.8%) and *n* = 86 males (42.2%), and participants’ ages ranged from 14 to 19 years (M = 15.88, SD = 1.43). Based on previous research [34,35,37], the sample was divided into three age groups: early adolescence (14–15 years; *n* = 101, 49.51% of the sample), mid-adolescence (16–17 years; *n* = 68, 33.33%), and late adolescence (18–19 years; *n* = 35, 17.16%). All participants subscribed to at least one social network, with the most used being Instagram (96.7%), followed by YouTube (85.5%), TikTok (78.9%), Facebook (40.2%), Twitter (30.9%), Snapchat (26.5%), and others (4.9%). Most of the adolescents participating in the study (91%) reported that their family belonged to a middle socioeconomic class [38].

No special inclusion and/or exclusion criteria were used for participation. The sampling technique used was convenience sampling; the sample is consequently not representative of the entire population. This type of sampling allows for generalization, that is, it ensures that the knowledge gained is representative of the population from which the sample was drawn [39,40].

All adolescents were provided a written informed consent form to sign; in most cases, as they were minors, their parents or guardians signed and consented to their participation. All subjects agreed to participate anonymously in the present study through an online questionnaire, and all subjects answered all the questions that were administered to them; consequently, the percentage of removed or missing data was 0%.

### 2.2. Measures

Body image perception was assessed using the Body Image Concern Inventory (BICI), a self-report measure designed to assess dysmorphic preoccupation [32]; it consists of 19 items rated on a 5-point Likert scale (from 1 “never” to 5 “always”) regarding dissatisfaction and preoccupation with physical appearance, control and camouflage behavior, reassurance-seeking, social concerns, and appearance-related avoidance. In the original study of the English language version [41], the BICI scores included the entire possible range (19–95, M = 50.4, SD = 14.2); factor analysis yielded two interpretable factors, dysmorphic symptoms and symptom interference, which explained 51.8% of the total variance. The Italian version of the BICI (I-BICI) used in this study showed good internal consistency, with a Cronbach’s α = 0.815 [42]; in this study, Cronbach’s α was 0.93 (α = 0.92 for the “dysmorphic symptoms” factor and α = 0.75 for the “symptom interference” factor).

Considering the very well established use of this instrument, the Bergen Instagram Addiction Scale (BIAS) was used to examine social media use, in particular Instagram. The BIAS was developed by adapting the Bergen Social Media Addiction Scale (BSMAS) [43], a six-item self-report questionnaire developed to measure the six core characteristics of social media addiction: salience, mood modification, tolerance, withdrawal, conflict, and relapse [44]. The BIAS, on the other hand, consists of 18 items, three for each of the six core characteristics of addiction, that relate to experiences over a 12-month period. The items are rated on a five-point Likert scale from 1 (very rarely) to 5 (very often); higher scores indicate greater Instagram addiction [45]. The Cronbach’s α detected in this research related to all items is α = 0.92, while that related to individual scales is as follows: α = 0.78 for the salience scale, α = 0.84 for the tolerance scale, α = 0.79 for mood modification, α = 0.81 for relapse, α = 0.83 for withdrawal scale, and α = 0.66 for conflict scale.

One of the most widely used instruments in the literature to observe internet use is the Internet Addiction Test (IAT) [46], a 20-item scale in which respondents are asked to rate on a five-point Likert scale (where 1 is “never” and 5 is “always”) the extent to which internet use affects their daily routine, social life, productivity, sleep, and feelings. The minimum score is 20 and the maximum is 100; the higher the score, the more problems are caused by internet use. Young [46] suggests that a score between 20 and 39 is typical of an average Internet user who has complete control over their internet use, while a score between 40 and 69 indicates frequent problems due to internet use and a score between 70 and 100 means that the internet is causing significant problems. This test showed strong internal consistency (α = 0.90–0.93) and good test–retest reliability (r = 0.85) [47,48]. The Italian version of the IAT was used in this study [49], and the Cronbach’s α obtained was α = 0.86.

The Youth Self Report (YSR) [50] is a self-descriptive instrument for the purpose of obtaining direct information from individuals in the 11–18 age group on behavioral and emotional skills and problems. By responding to 112 items in the form of statements about behaviors in various domains and emotional problems, adolescents provide an account of themselves, their adaptive functioning, and their emotional–behavioral characteristics over the past six months, using a three-point response scale: 0 (not true or not at all), 1 (sometimes or somewhat true), and 2 (very true or often). The instrument presents nine syndromic scales: anxiety/depression, withdrawal/depression, somatic complaints, social problems, intrusive thoughts, attention problems, rule-breaking behaviors, aggressive behaviors, and other problems. Syndromes are further distinguished into total, internalizing, and externalizing problems [29,30]. Standardized and cut-off scores allow comparison between clinical and non-clinical populations, with good reliability and validity values; the YSR scale is a checklist characterized by good reliability and validity (range = 0.66-.87) in different cultural and linguistic contexts [51,52,53]. In the present research, the Cronbach’s α obtained was excellent (α = 0.92): α = 0.88 for the internalizing problem scale and α = 0.84 for the externalizing problem scale.

### 2.3. Data Analyses

Data analyses included descriptive statistics (means and standard deviations) and correlations between the variables of interest for the total sample, for each gender, and for each age group. Using analysis of variance (ANOVA), it was possible to observe differences by gender and by age in every variable of interest. In addition, using analysis of covariance (ANCOVA), it was possible to control for confounding factors, in this case age and gender.

Bivariate correlations were used, and Pearson’s r coefficient was calculated to analyze the associations between BIC, SMA, and IA. In addition, after checking for significant correlations between the investigated variables and to see whether IA mediated SMA, a multiple regression analysis was performed by including first gender and then age as variables to examine the predictive associations between body perception and social network and internet addiction.

A *p* value of less than 0.05 was generally considered statistically significant in the analyses.

## 3. Results

For each questionnaire administered (I-BICI, BIAS, IAT, YSR), total and subscale scores were calculated along with the Cronbach’s α coefficients shown above. The means and standard deviations of the raw scores of the items of the study variables obtained from both the sample and subgroups divided by gender and age are shown in Table 1.

### 3.1. Differences among Subjects by Gender and Age

Using ANOVA, we performed between-group comparisons; gender differences in body image satisfaction were expected, as women generally report lower satisfaction with their physical appearance and higher concerns about appearance and weight [46]. Indeed, the results of the one-way ANOVA related to the I-BICI revealed significant differences by gender on levels of dysmorphic concern (F (1, 202) = 47.284, *p* < 0.01), social media use (F (1, 202) = 8.364, *p* = 0.004), and internet use (F (1, 202) = 7.799, *p* = 0.006). In addition, concerning YSR, gender differences were observed in the internalizing problems scale (F (1, 202) = 27.139, *p* < 0.01). ANOVA showed no significant age-related differences in the observed variables.

However, ANCOVA showed that although the model for each variable considered in the study was statistically significant (respectively, R^2^ = 0.192 for I-BICI, R^2^ = 0.048 for SMA, R^2^ = 0.040 for IA, R^2^ = 0.141 for internalizing symptoms, and R^2^ = 0.068 for externalizing symptoms), when controlling for confounding factors, gender was statistically significant for all variables except for externalizing symptoms ((F (1, 201) = 2.746, *p* = 0.099), while age was statistically significant only for internalizing problems ((F (1, 201) = 5.242, *p* = 0.023) and externalizing problems ((F (1, 201) = 13.428, *p* < 0.01).

### 3.2. Correlations between the Variables of Interest Considering the Total Sample and Subgroups Related to Gender and Age

Pearson correlations between I-BICI, BIAS, IAT, and YSR scores were calculated for the total sample and subgroups. Results for the total sample indicated that all variables of interest were positively and significantly correlated with each other, as can be seen in Table 2.

Regarding gender-related correlations, the results are shown in Table 3. While the Pearson’s coefficient values are positive and in line with those observed in the total sample, it is interesting to note the strong association found in males between I-BICI and IAT and between I-BICI and the YSR internalized symptom scale, which was greater than in females.

Finally, Table 4 shows the bivariate correlations for each age group. It is interesting to note that in the early adolescence group, all correlations are significant except those between BIAS and YSR for internalizing and externalizing symptoms. For the 16–17 age group, all correlations are significant except those between externalizing symptoms measured with the YSR, which are significantly correlated only with the I-BICI. Finally, late adolescents differ from the rest of the sample; in fact, only internet use (IAT) and social use (BIAS), I-BICI with internalizing symptoms, and BIAS and YSR externalizing symptoms are found to be significantly correlated.

### 3.3. Association of Technological Addictions (IA and SMA) with Body Image Concerns

As was expected, the bivariate correlations between BIAS and IAT proved to be strong, thus, to test the mediation of IA on SMA, a multiple regression analysis was performed for the total sample and gender- and age-related subgroups.

Regarding the total sample, the results showed a significant correlation between I-BICI and IAT and between I-BICI and BIAS: this pattern explains 16.7% of the variance (R^2^ = 0.167), which is statistically significant, F (2, 201) = 20.176, *p* < 0.01. Notably, the IAT is positive and significant (β = 0.219, t (201) = 2.382, *p* = 0.018), as is the BIAS (β = 0.223, t (201) = 2.426, *p* = 0.016).

Running a multiple regression by gender, the IAT was significant for males (β = 0.383, t (83) = 2.659, *p* = 0.009), while it was non-significant for females (β = 0.110, t (115) = 0.908, *p* = 0.366), while the regression coefficient associated with BIAS was significant for girls (β = 0.242, t (115) = 1.995, *p* = 0.048) and not for boys (β = 0.081, t (83) = 0.564, *p* = 0.574).

Regarding age, the pattern for adolescents who were part of the third group (18–19 years) was nonsignificant (R^2^ = 0.106, F (2,32) = 1.894, *p* = 0.167). The IAT was statistically significant for both the 14–15 year age group (β = 0.243, t (98) = 2.028, *p* = 0.045) and the 16–17 year age group (β = 0.387, t (65) = 2.224, *p* = 0.030); finally, the regression coefficient associated with BIAS was non-significant for both the former group (β = 0.212, t (98) = 1.767, *p* = 0.080) and the latter (β = 0.119, t (65) = 0.683, *p* = 0.497).

## 4. Discussion

### 4.1. Main Findings

The purpose of the present study was to investigate adolescents’ use of the Internet and social media and their concerns about their bodies, as well as to observe the presence of psychopathological risks (internalizing or externalizing problems). The objective was to investigate the possible association between these variables. In fact, the results of previous meta-analyses [21,28,54] have indicated that the massive presence of appearance-related content on the internet and excessive use of social networks could represent a new socio-cultural predictor of body dissatisfaction; young adults’ preference for new media could therefore explain the impact that the internet and social media have on the possible development of appearance-related problems.

As males and females have been found to have different characteristics in their use of the internet and social networks [55], the total sample was divided by gender. In this regard, it was observed that while females have a greater vulnerability to internalizing problems such as anxiety and depression [56,57], males usually express their distress with externalizing symptoms [56]. The sample was then divided by three age groups, early, middle, and late adolescence, following Steinberg’s suggestions [36].

The results of the present study demonstrated the existence of a correlation between SMA and BIC: this correlation was significant, positive, and moderate for both the total sample and the gender and age subgroups. The only group in which no significant correlation was observed was late adolescence (r = 0.319, *p* = 0.062). The same was observed in the correlation between IA and BIC; correlations were significant, positive, and moderate for both the total sample and the various groups, with the only exception again being late adolescence (r = 0.190, *p* = 0.274). These results are in line with previous studies indicating that excessive use of social media can have a negative impact on body image [27,58,59,60,61,62,63,64].

Interestingly, although it would appear from the ANOVA results that girls are more dissatisfied with their physical appearance than boys, the correlations show instead that boys are more influenced by social media (as observable in Table 3). This contradicts previous studies (e.g., [65,66,67]) in which it was shown that women are more motivated to use social media to compare themselves with others and are more influenced by it to adapt and improve their online self-image and self-presentation. It is likely that previous research focused only on the female population, and therefore may not accurately reflect SMA and gender differences; in fact, results from studies that include male samples have proven contradictory [68,69,70].

The present study showed that in both the total sample and in the gender and age subgroups, IA appears to have a greater association, albeit slightly, with body-related concerns than SMA. This is probably due to the fact that SMA is part of the more general IA, which is therefore a more significant source of influence, as confirmed by multiple regression. The association between internet use and body dissatisfaction observed in this study reflects the results of previous research [16,66,71]. However, again, these studies primarily involve female samples; there is little research that includes the male gender or involves exclusively male samples.

Another objective of this study was to observe the possible association with internalizing or externalizing psychopathological risks. It is interesting to note the significant and positive association found between internalizing problems and BIC in both the total sample and in the subgroups (range 0.366–0.609). These results can be interpreted within the theoretical framework of Social Comparison Theory [72]; as with mass media in general, network content becomes a source of comparison for both boys and girls. More frequent use and interaction thus increase opportunities to confront the types of images that often portray a socially accepted bodily ideal [62]. The perceived gap between one’s assessment of one’s body image and what is posted on the internet fosters an increase in dissatisfaction and concerns about physical appearance, which in turn makes the development of internalizing symptoms, such as depression and anxiety, more likely [63]. However, as this is a cross-sectional study, it is not possible to determine whether BIC is a predictor of internalizing symptoms; according to Aderka and colleagues [73], it is plausible that internalizing symptoms play a role in predicting BIC. On the contrary, considering a different perspective [37], longitudinal studies have shown that internalizing symptoms may precede increased social media use [74,75,76]; and social media use was even associated with reduced psychological distress thanks to the social opportunities provided by social networking sites [77].

Finally, division by age group allowed us to observe differences during this developmental stage. Specifically, regarding the association between BIC and technology addictions (IA and SMA), the strongest associations occurred in middle adolescence (r = 0.480 and r = 0.421, respectively, *p* < 0.01) followed by early adolescence (IAT, r = 0.379; SMA, r = 0.368), while in the late adolescent group the associations were not significant. However, it should be considered that the late adolescent group was less numerous than the others (*n* = 35), and thus the results obtained in this research may not be representative. In fact, previous studies (e.g., [62,78,79,80]) focusing on this age group and young adults have observed social media use to have a negative effect on BIC and body dissatisfaction.

Regarding internalizing and externalizing symptoms, similar results were observed in the associations of the variables of interest: the group with the highest number of significant correlations (moderate and positive) is middle adolescence, followed by early adolescence; again, late adolescence differs from the other groups, obtaining only two significant associations (body image concern and internalizing problems: r = 0.410, *p* < 0.05; IA-externalizing problems: r = 0.480, *p* < 0.01).

From the point of view of psychopathology [64], the findings in younger adolescent subgroups may be explained by desire for greater independence, greater attention to social interactions and friendships, and greater emotional distance from parents. At this developmental stage, feelings of inferiority towards other individuals, discomfort in interpersonal interactions, low self-esteem, and lack of assertiveness may be specific manifestations of interpersonal sensitivity [81]. Therefore, excessive use of the internet and social media could be a strategy to escape these negative feelings.

The results obtained in the present study corroborate previous literature [34,35] showing that 16–17-year-old adolescents are characterized by higher levels of psychopathological risk, especially internalizing risk, associated with higher levels of SMA and more complex psychological functioning than other subgroups. However, these relationships are controversial and may change depending on the developmental stage (early, middle, and late adolescence) and sex of the individual [82,83].

### 4.2. Limitations and Suggestions for Future Research

This study has several limitations. First, as it is not a longitudinal study, it does not allow for causal hypotheses. Second, as it is a cross-sectional sample study, it is culturally limited to young adolescents who grew up in Italy and does not allow the results to be extended to all cultures, although they seem in line with the previous literature. Finally, this study did not consider potentially confounding variables (e.g., personality traits, presence of medical conditions, and other biological and cultural factors, including internet exposure).

Future research should aim to expand the samples used in this and previous studies in order to investigate gender and age differences; the association with male body dissatisfaction, in particular, merits further investigation, especially considering the increased presence in the media of images related to fitness and the muscular ideal that could lead to the development of muscle dysmorphia, a problem typically associated with male adolescents. Furthermore, in light of the serious health and well-being consequences described in this manuscript, the idea of a prospective public health cohort study examining adolescents in communities with very limited or no access to the internet and social media and adolescents who do have extensive access to it, sampled over time using the tools mentioned in this study and others, is warranted.

## 5. Conclusions

The results of this paper contribute to the growing number of studies examining the relationship between technology addictions (IA and SMA) and body image. Considering the increasing use of these technologies, especially by adolescents, it is critical to develop an understanding of how these relationships work. Indeed, as the results of this study and the previous literature suggest, all adolescents, both male and female, are affected by new media.

Despite research moving in this direction, this is, to our knowledge, the first study to consider age (divided into early, middle, and late adolescence) as a variable; in fact, most of the previous literature focuses on young adults. The results obtained in this work underscore the need to investigate the entire period associated with adolescence and to focus on preadolescents and early adolescents as well; IA and SMA would appear to have an impact on body image, which is undergoing formation during this period [7,10,14].

Indeed, although this is a cross-sectional study, it is important to consider that common features of the internet and social media could explain their association with the development of body dissatisfaction, as these mediums provide constant access to image-centered content. Examining social engagement on social media and the internet may be a key factor in understanding how they influence people’s average satisfaction with their bodies and appearance.

## Figures and Tables

**Table 1:** Descriptive statistics (mean and standard deviation) for the variables of interest.

		Females *n* = 118	Males *n* = 86	Total *n* = 204	14–15 Years *n* = 101	16–17 Years *n* = 68	18–19 Years *n* = 35
**BODY** **IMAGE** **CONCERNS**	Dysmorphic Symptoms	47.14 (11.82)	35.97 (9.82)	42.43 (12.31)	41.58 (11.26)	41.90 (13.08)	45.89 (13.40)
	Symptom Interference	7.37 (3.40)	5.79 (2.20)	6.71 (3.05)	6.51 (2.50)	6.34 (3.28)	7.97 (3.76)
	Total	54.50 (14.33)	41.76 (11.14)	49.13 (14.5)	48.10 (12.78)	48.24 (15.52)	53.86 (16.48)
**SOCIAL** **MEDIA** **ADDICTION**	Salience	6.70 (2.73)	6.29 (2.67)	6.51 (2.70)	6.51 (2.69)	6.50 (2.61)	6.54 (2.96)
	Tolerance	6.89 (3.07)	5.97 (2.66)	6.50 (2.94)	6.03 (2.89)	6.94 (2.82)	7.00 (3.15)
	Mood Modification	5.99 (2.92)	5.24 (2.41)	5.68 (2.74)	5.43 (2.41)	5.69 (2.81)	6.37 (3.39)
	Relapse	5.76 (2.98)	4.60 (2.39)	5.27 (2.80)	4.81 (2.19)	6.13 (3.41)	4.91 (2.77)
	Withdrawal	4.85 (2.35)	4.15 (2.25)	4.55 (2.33)	4.27 (2.12)	4.72 (2.25)	5.06 (2.93)
	Conflict	5.63 (2.73)	4.63 (1.59)	5.21 (2.37)	4.98 (2.11)	5.38 (2.24)	5.51 (3.18)
	Total	35.78 (12.69)	30.88 (10.82)	33.71 (12.15)	32.10 (10.97)	35.37 (12.66)	35.40 (13.98)
**INTERNET** **ADDICTION**		43.65 (10.65)	39.51 (10.18)	41.91 (10.63)	41.28 (9.13)	41.78 (11.12)	43.97 (13.42)
**EMOTIONAL–BEHAVIORAL** **FUNCTIONING**	Internalizing Problems	20.64 (9.08)	14.33 (7.74)	17.98 (9.08)	16.75 (8.77)	17.47 (8.89)	22.49 (9.16)
	Externalizing Problems	10.80 (5.85)	11.77 (7.33)	11.21 (6.53)	9.51 (5.74)	12.79 (7.21)	13.00 (6.19)
	Total	51.59 (19.79)	46.06 (20.39)	49.26 (20.18)	45.60 (19.70)	50.04 (19.64)	58.29 (20.15)

**Table 2:** Bivariate correlations among the variables of interest for the total sample.

	BIC	SMA	IA	INT	EXT
**BIC**	-	0.379 **	0.378 **	0.519 **	0.238 **
**SMA**	-	-	0.714 **	0.204 **	0.156 *
**IA**	-	-	-	0.287 **	0.220 **

* p < 0.05; ** *p* < 0.01. BIC = Body Image Concerns; SMA = Social Media Addiction; IA = Internet Addiction; INT = Internalizing Problems; EXT = Externalizing Problems.

**Table 3:** Bivariate correlations among gender-related variables of interest.

	BIC	SMA	IA	INT	EXT
**Females**					
**BIC**	-	0.318 **	0.276 **	0.366 **	0.280 **
**SMA**	-	-	0.687 **	0.127	0.198 *
**IA**	-	-	-	0.164	0.260 **
**Males**					
**BIC**	-	0.361 **	0.442 **	0.585 **	0.349 **
**SMA**	-	-	0.730 **	0.185	0.151
**IA**	-	-	-	0.368 **	0.220 *

* p < 0.05; ** *p* < 0.01. BIC = Body Image Concerns; SMA = Social Media Addiction; IA = Internet Addiction; INT = Internalizing Problems; EXT = Externalizing Problems.

**Table 4:** Bivariate correlations between age-related variables of interest.

	BIC	SMA	IA	INT	EXT
	**14–15 Years**				
**BIC**	-	0.368 **	0.379 **	0.465 **	0.197 **
**SMA**	-	-	0.642 **	0.181	0.104
**IA**	-	-	-	0.248 *	0.215 *
	**16–17 years**				
**BIC**	-	0.421 **	0.480 **	0.609 **	0.309 *
**SMA**	-	-	0.782 **	0.280 *	−0.021
**IA**	-	-	-	0.315 **	0.168
	**18–19 years**				
**BIC**	-	0.319	0.190	0.410 *	0.123
**SMA**	-	-	0.736 **	0.068	0.480 **
**IA**	-	-	-	0.271	0.303

* *p* < 0.05; ** *p* < 0.01. BIC = Body Image Concerns; SMA = Social Media Addiction; IA = Internet Addiction; INT = Internalizing Problems; EXT = Externalizing Problems.

## Data Availability

Data is available on request to the corresponding author.
